# First patient in the Iranian Registry with novel DOCK2 gene mutation, presenting with skeletal tuberculosis, and review of literature

**DOI:** 10.1186/s13223-021-00631-5

**Published:** 2021-12-06

**Authors:** Niusha Sharifinejad, Homa Sadri, Arash Kalantari, Samaneh Delavari, Amirhosein Noohi, Yasaman Aminpour, Araz Sabzevari, Gholamreza Azizi

**Affiliations:** 1 Non-Communicable Diseases Research Center, Alborz University of Medical Sciences, Karaj, Iran; 2 Department of Immunology and Allergy, Imam Khomeini Hospital, Tehran University of Medical Sciences, Tehran, Iran; 3 Research Center for Immunodeficiencies, Pediatrics Center of Excellence, Children’s Medical Center, Tehran University of Medical Science, Tehran, Iran; 4 Department of Pediatrics, Imam Ali Hospital, Alborz University of Medical Sciences, Karaj, Iran; 5 CinnaGen Medical Biotechnology Research Center, Alborz University of Medical Sciences, Karaj, Iran

**Keywords:** Primary Immunodeficiency, Dedicator of cytokinesis 2 deficiency, DOCK2 deficiency, Combined immunodeficiency, CID

## Abstract

**Background:**

Dedicator of cytokinesis 2 (DOCK2) deficiency is an inborn error of immunity characterized by cellular and humoral immunological abnormalities leading to early-onset infections.

**Case presentation:**

We reported a novel case of a 27 months old girl presenting with recurrent pneumonia and a history of skeletal tuberculosis at the age of 19-month-old. Her immunological workup revealed persistent lymphopenia and low CD4 + T cell count along with elevated levels of CD19 +, CD20 +, CD16 +, and CD56 + cells. Furthermore, she had a high level of immunoglobulin (Ig) E and a slightly reduced IgM level with a non-protective antibody titer against diphtheria. The whole-exome sequencing (WES) analysis identified a homozygous frameshift deletion mutation (c.1512delG, p.I505Sfs*28) in exon 16 of the *DOCK2* gene. We also conducted electronic searches in PubMed, Web of Science, and Scopus databases and reviewed the articles reporting patients with DOCK2 deficiency. The literature search yielded 14 DOCK2-deficient patients suffering from both cellular and humoral immune defects leading to early-onset infections, particularly human herpesvirus (HHV) infection.

**Conclusion:**

DOCK2 deficiency should be considered in the context of severe or unusual early-onset infections, especially HHV infections, in a patient with a probable clinical diagnosis of combined immunodeficiency. We also recommended that DOCK2-deficient patients might benefit from T-cell receptor excision circle (TREC) assay as part of the routine newborn screening program.

## Introduction

Dedicator of cytokinesis 2 (DOCK2) is a subfamily of guanine exchange factors required for the activation of intercellular GTPases and subsequent release of adenosine triphosphate (ATP) in response to various stimuli [1]. DOCK2, specifically, expresses on immune cells and promotes the activation and migration of B cell and T cell lymphocytes. Besides, it promotes the cytotoxicity and degranulation of natural killer (NK) cells and the thymic development of NKT cells. Additionally, DOCK2 is reported to mediate the production of reactive oxygen species (ROS) and chemotaxis in neutrophils [2].

Biallelic loss-of-function mutations in the *DOCK2* gene are associated with clinical features of combined immunodeficiency (CID) according to the primary immunodeficiency (PID) treatment consortium (PIDTC) [3, 4]. Different *DOCK2* mutations resulted in absent or significantly reduced levels of DOCK2 protein expression that precede a chain of immunological defects including CD4 + and CD8 + T cells lymphopenia with reduced T cell receptor excision circles (TRECs), diminished phytohemagglutinin (PHA)-induced T cell proliferation, impaired NK cell function, decrease B cell counts, and defective antibody responses in DOCK2-deficient patients [3]. These immunological disturbances commence early-onset and severe infections in patients harboring *DOCK2* mutations that often lead them to death [5]. Hematopoietic stem cell transplantation (HSCT) is currently the only curative option for DOCK2-deficient patients [3].

Here, we report a 27 months old girl who presented with severe infection and carried a novel homozygous frameshift deletion c.1512delG (p.I505Sfs*28) mutation at exon 16 of the *DOCK2* gene*.* We have also reviewed the data of previously reported patients with *DOCK2* deficiency.

## Patient and methods

The demographic data, medical history, and physical examination of the patient were obtained through direct interviews and examining the patient’s clinical record in the Imam-Ali Karaj Hospital affiliated to Alborz University of Medical Sciences, using national consensus on diagnosis and management guidelines for PID [6]. Written informed consent was obtained from the parents, following the principles of the ethics committee of the Alborz University of Medical Sciences. Basic hematological, biochemistry, and immunological investigations including enumeration of lymphocyte subsets: T- and B-cells subsets (assessed using flow cytometry analysis), serum levels of immunoglobulins, complements, and antibodies (assessed using nephelometry and enzyme-linked immunosorbent assay).

Secondary causes of lymphopenia were excluded by history taking and laboratory tests, and no sign of drug or disease-related causes was detected. Clinical diagnosis of CID has been established according to the criteria of the European Society for Immunodeficiencies [7]. The whole peripheral blood sample was used to extract genomic DNA. The whole-exome sequencing (WES) and the confirmatory Sanger sequencing were performed according to a previously published pipeline [8, 9].

The literature searches for reported DOCK2-deficient patients were conducted in PubMed, Web of Science, and Scopus, using the following keywords: DOCK2 deficiency and Dedicator of cytokinesis 2 deficiency. The articles were primarily screened based on the title and abstract to exclude immaterial studies and then the full-text manuscripts of the included studies were assessed to determine their eligibility criteria (written in English, conducted on human subjects, reporting at least one patient with DOCK2 deficiency diagnosis). The descriptive section is subsequently developed based on this information.

### Case presentation

**Fig. 1:**
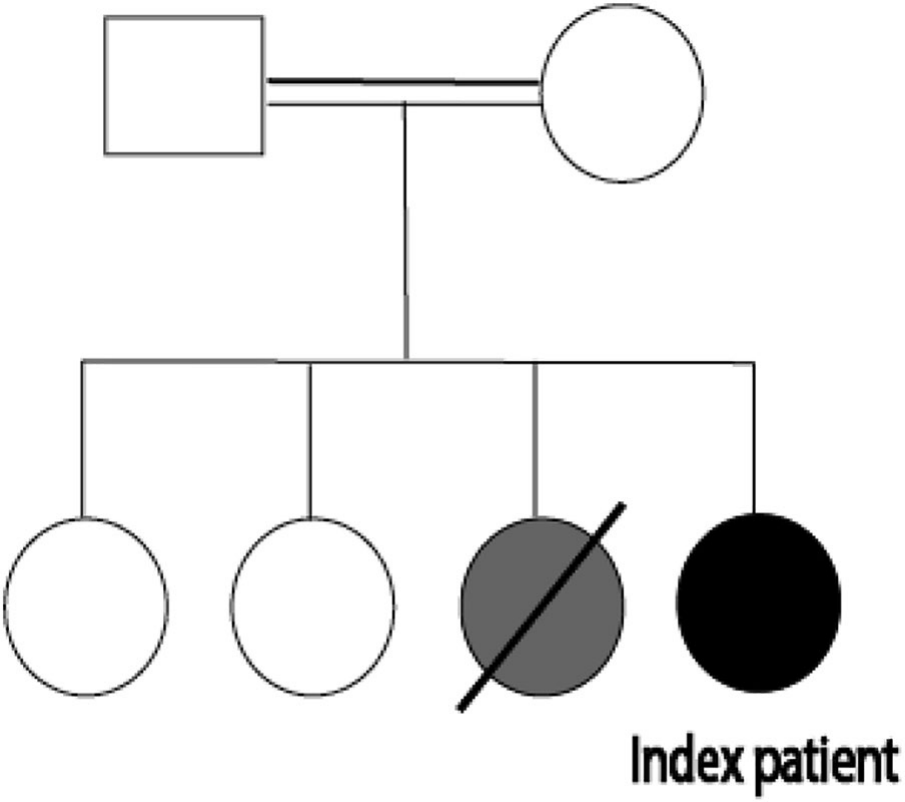
The family pedigree of the patient with *DOCK2* deficiency

**Fig. 2:**
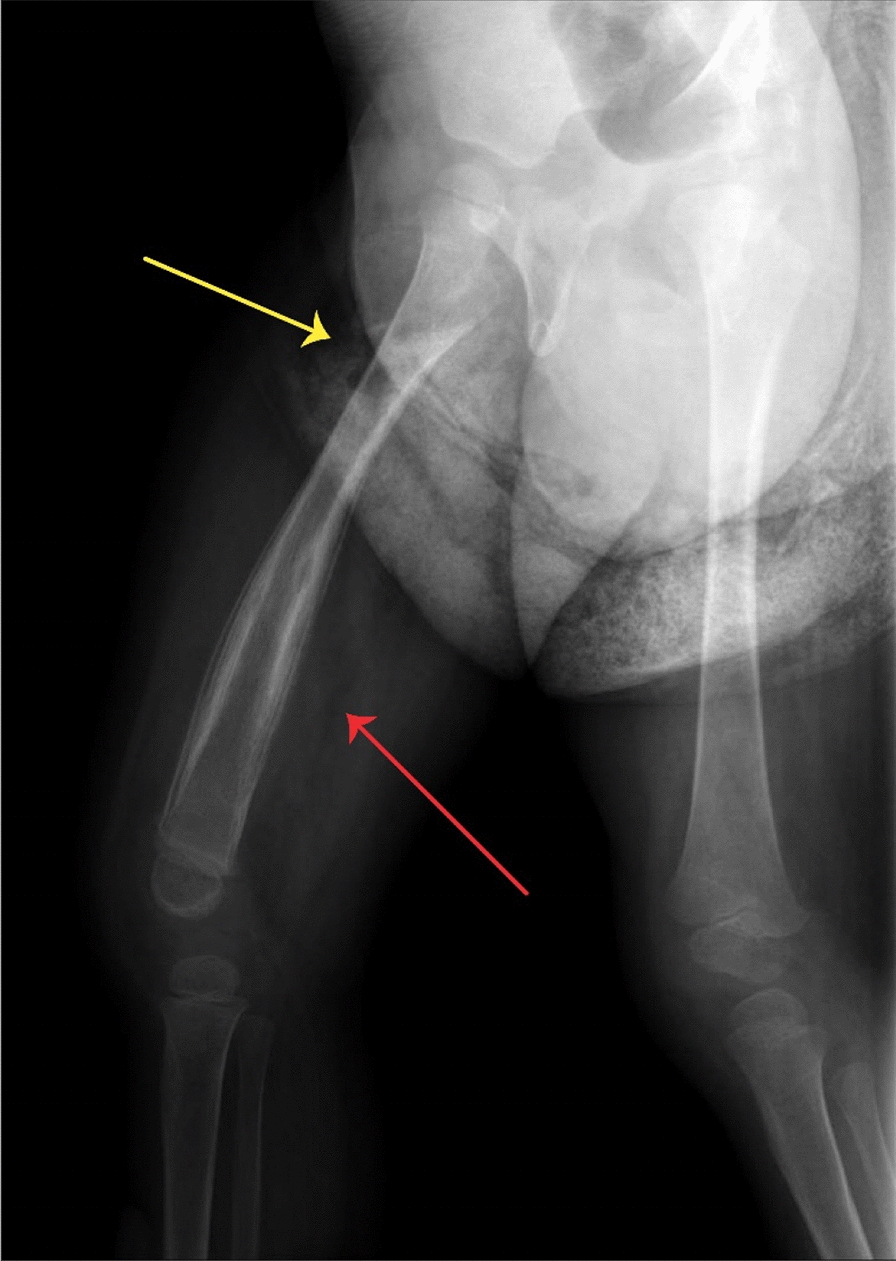
The X-ray of the patient shows signs of periosteal reaction in the diaphysis (red arrow) and a lucent area with sclerotic borders in the metaphysis of right femur (yellow arrow)

The patient was a 26-month-old girl born to first-degree consanguineous Iranian/Turkish parents. She was born at term gestational age with 3200 g weight and underwent routine age-matched vaccination in Iran including BCG vaccine at birth, Polio, Hepatitis B, Diphtheria, Tetanus, Haemophilus Influenza type B, Pertussis, and MMR (mumps, measles, rubella) until 18 months old, without remarkable adverse event. She had an older sister who died at the age of 1.5 years with diarrhea as the main clinical symptom (Fig. 1). At 18 months of age, she was hospitalized due to pneumonia and otitis media with perforated tympanic membrane and pus discharge. By that time, her growth and developmental state were age-appropriate and she had not experienced prior hospitalization or considerable infection. Only a few days after her discharge, she developed right thigh swelling, tenderness, and heat, unable of weight-bearing. Therefore, she was admitted once more for suspected acute osteomyelitis. The right femur X-ray showed evidence of osteomyelitis (Fig. 2). Later, she underwent an open biopsy and the mycobacterium tuberculosis (MTB) DNA complex was detected in the tissue sections obtained from her right femur, using Xpert MTB/RIF Ultra assay which is a rapid and accurate diagnostic tool, especially in extra-pulmonary tuberculosis [10]. Plus, the smear of skeletal canal fluid specimen confirmed the presence of many acid-fast positive bacilli. Meantime, chest radiographs of the patient and her parents were evaluated and although her parents had no lesion compatible with pulmonary TB, the patient’s X-ray showed round density in the middle lobe of the right lung. They had no contact history of TB. She was treated with Isoniazid, Rifampin, Ethambutol, Pyrazinamide, and vitamin B6 for 9 months with the diagnosis of skeletal tuberculosis. The patient’s unusual infection with an uncommon microorganism prompted an immunological workup to detect the possible underlying immunodeficiency (Table 1). Based on her clinical and initial laboratory data, she was recommended for a thorough evaluation, but her parents refused further investigation.

**Table 1:** Immunologic work-up of the DOCK2 deficient-patient

Parameters	19 months old	26 months old	Normal ranges
WBC × 10^3^ (cell/µL)	**25.5**	6.7	5.5–15.5
Lymphocytes (cell/µL )	**11,700**	**1641**	3873–6141
Hemmoglobin (g/dL)	**9.5**	**9.8**	10.9–15
CD3 + T cells (cell/µL)	**6470**	1709	1578–3707
CD4 + T cells (cell/µL)	**322**	**34**	870–2144
CD8 + T cells (cell/µL)	**2697**	581	472–1107
CD4/CD8 Ratio	**0.16**	**0.058**	0.9–3.7
CD19 + (cell/µL)	**2597**	**1900**	434–1274
CD20 + (cell/µL)	ND	**1900**	124–1665
CD16 + (cell/µL)	1111	**1178**	78–703
CD56 + (cell/µL)	**1743**	**874**	45–555
CD16 + 56 (cell/µL)	**760**	380	155–565
IgG (mg/dL)	ND	511	424–1051
IgM (mg/dL)	79	**41**	48–168
IgA (mg/dL)	72	85.1	14–123
IgE (IU/mL)	**471**	**400**	< 100
C3 (mg/mL)	113	ND	84–174
C4 (mg/mL)	27	ND	12–40
CH50 (IU/mL)	> 90	116	70–150
MFI/PMA ox-DHR (%)	40.2%	ND	> 30%
HIV 1,2 antigen/ antibody (S/CO)	ND	0.03	< 0.9
Anti-tetanus antibody (IU/mL)	ND	0.19	> 0.1
Anti-diphtheria antibody (IU/mL)	ND	**0.03**	> 0.1

*ND* not determined, *Ig* immunoglobulin, *WBC* white blood cell, *C3* complement component 3, *C4* complement component 4, *CH50* total hemolytic complement, *MFI/PMA ox-DHR* mean fluorescence intensity/phorbol myristate acetate oxy dihydrorhodamine, *HIV 1,2* human immunodeficiency virus 1,2

Abnormal value are indicates in bold

A few months later, she was referred to our hospital with fever, cough, and shortness of breath and eventually admitted to the pediatric intensive care unit (PICU) due to severe tachypnea and respiratory distress with the diagnosis of severe pneumonia. During her admission, she received empirical broad-spectrum antibiotics with full coverage of bacteria, viruses, and fungi. Nonetheless, no pathogenic microorganism was isolated from the patient and despite receiving the standard treatment, her respiratory condition worsened and she got intubated.

Her inadequate response to standard treatment led to afresh immunological workup. The laboratory results revealed anemia, lymphopenia along with decreased CD4 + count, and CD4/CD8 ratio similar to her previous records (Table 1). She also had increased CD19 +, CD20 +, CD16 +, and CD56 + cell counts. Her anti-diphtheria antibody titer was beneath the protective level despite the age-matched vaccination. She had hyper immunoglobulin (Ig) E and a slightly reduced IgM level (Table 1). Based on her results, a probable CID was suspected and genetic analysis was performed on whole blood samples using WES. The WES analysis and Sanger sequencing revealed a homozygous frameshift deletion mutation c.1512delG (p.I505Sfs*28) at exon 16 in the *DOCK2* gene loci*.*

Meanwhile, her serum creatinine and urea level increased significantly (2.5 and 248.5 mg/dL, respectively) and she underwent peritoneal dialysis. During her admission, she suffered from pneumothorax and few episodes of seizure as well. Unfortunately, she was deceased at the age of 27-month-old due to multi-organ failure.

## Review of literature

So far, 14 DOCK2-deficient cases (8 males, 4 females, and 2 unknown gender) were reported in six different studies from various ethnicities. Consanguinity and positive family history of immunodeficiency were reported in 81.8% (9 out of 11 cases) and 44.4% (4 out of 9 cases) of patients, respectively. All patients with available data developed infection as their first manifestation of immunodeficiency within the first months of birth. Half of the patients (6 of 12) also presented with infections caused by different members of the human herpesvirus family (HHV) including HHV6, varicella, and *Cytomegalovirus* (CMV). Fifty-seven percentage (8 of 12) of patients had lymphopenia. The major immunological abnormalities in lymphocyte subsets were; low CD3 + in 64.2% (9 of 14), low CD4 + in 78.6% (11 of 14), low CD8 + in 50% (7 of 14), low CD19 + in 50% (7 of 14), and low NK counts in 50% (7 of 14).

IgM levels were decreased in 7 cases (50%) and increased in 3 cases (21.4%). Elevated/or reduced IgA levels were present in 3 cases (21.4%). IgG levels were mainly normal among the patients (78.6%). Nine patients showed decreased T-cell response to PHA, and of those, six patients had the SCID response range [11]. All of the cases that were tested for TREC (4 cases) had decreased TREC levels. Poor response to vaccination and absent antibody responses to infectious agents were also reported in some cases. HSCT was performed as the main treatment in 77.8% (7 of 9) of patients resulting in achieved engraftment in 5 (71.4%) patients. Further information is available in Table 2.

**Table 2:** An overview of the clinical and immunological findings of reported cases with DOCK2 deficiency

No	Sex	Ethnicity	CON	FH	AOO (m)	AF-Inf	Infection	Other manifestations	Immunological abnormality	*DOCK2* mutation/protein	Treatment	Outcome	Refs.
1	M	Lebanese	+	−	3	3	RSV bronchiolitis, recurrent pneumonia,	–	Lymphopenia, decreased CD3 +, CD4 + , and CD8 +, decreased IgM level, decreased T-cell response to PHA	p.Y1242Yfs*33	HSCT (myeloablative)	Alive	[3]
2	F	Finnish	−	−	< 24	< 24	Recurrent otitis media, pneumonia, diarrhea, Varicella, *M. avium,* and HHV-6 infection	Three episodes of thrombocytopenia	Lymphopenia, decreased CD3 +, CD4 +, CD8 +, and CD19 + cells decreased IgM, increased IgE and IgA, decreased T-cell response to PHA, decreased TREC, Non-protective against tetanus toxoid, PRP, *Streptococcus pneumoniae*	p.R1104W, p.Q1324*	HSCT (reduced-intensity)	Alive	[3]
3	M	Turkish	+	+	3	3	Recurrent respiratory tract infections, meningoencephalitis, severe varicella infection, mumps,	–	Lymphopenia, decreased CD3 +, CD4 +, and CD8 +, decreased T-cell response to PHA, No response to VZV	p.R751S	ND	Dead	[3]
4	M	Turkish	+	−	< 3	< 3	Chronic diarrhea, oral moniliasis, recurrent pneumonia with *parainfluenza virus type 3* and *adenovirus*, CMV infection, *Klebsiella pneumoniae* sepsis	FTT, nodular erythematous lesion at the site of *bacille Calmette–Guerin* vaccination, hepatomegaly with persistently elevated aminotransferase levels, colitis	Lymphopenia, decreased CD3 +, CD4 +, CD19 +, and NK, decreased IgM level, increased IgA, decreased T-cell response to PHA, decreased TREC, Response to HBV not detectable	p.F744Cfs*27	ND	Dead	[3]
5	M	Hispanic	−	−	4	4	Interstitial pneumonia	Rectal fistula	Decreased CD3 +, CD4 +, CD8 +, increased NK cells, increased IgM and IgE, decreased T-cell response to PHA, Response to KLH not detectable	p.P1476L, p.M120Mfs*22	High-dose trimethoprim–sulfamethoxazole, HSCT (myeloablative)	Alive	[3]
6	F	Iranian	+	−	2	2	Septicemia, diarrhea, CMV infection	Seizures	Thrombocytopenia, lymphocytopenia, reduced CD4 +, CD19 +, NK cells, elevated IgM, decreased TREC, T cell response to PHA and T cell response to BCG	c.C3310T, p.R1104W	Antibiotics, antiviral treatment, and IVIG	Dead	[5]
7	M	Moroccan	+	+	0	0	*E.coli* pyelonephritis	Ulcerative perianal dermatitis, Omenn syndrome, nephrotic syndrome, ARDS, capillary leak syndrome	Lymphopenia, decreasedCD3 +, CD4 +, CD8 +, CD19 +, and NK, decreased IgM and IgA, Absent TREC, absent T cell response to PHA	c.2704-2 A > C	HSCT, etanercept, tocilizumab, and high-dose steroids	Dead	[12]
8	M	Moroccan	+	+	2.5	ND	Sepsis, Respiratory, distress, Hepatitis, CMV, *Enterovirus, Rhinovirus*, and *Pneumocystis jiroveci* infections	ARDS, Bloody diarrhea Livedo	Lymphopenia, decreased CD3 +, CD4 +, CD8 +, absent T cell response to PHA	c.2704-2 A > C	HSCT	Dead	[12]
9	F	Moroccan	+	+	0	ND	*Influenza A* and *Rhinovirus* infection	Bloody diarrhea	Lymphopenia, decreased CD3 +, CD4 +, CD8 +, CD19 +, and NK	c.2704-2 A > C	HSCT (without conditioning)	Alive	[12]
10	F	ND	+	ND	0.5	0.5	Recurrent sinopulmonary infections, CMV viremia	Chronic diarrhea	Decreased CD4 + and IgG level, increased IgM, decreased T cell response to PHA and BCG	c.del 902-1078	IVIG, ganciclovir, HSCT (myeloablative)	Alive	[13]
11	M	ND	+	ND	5	5	Recurrent pneumonias, oral candidiasis, sepsis	Chronic diarrhea	Decreased CD4 + and CD19 +, panhypogammaglobulinemia	Phe848fs	IVIG, prophylactic antibiotics	Dead	[13]
12	M	Indian	ND	ND	ND	ND	Otitis media, recurrent pneumonia with atypical mycobacterial and influenza Infections	–	Decreased CD3 +, decreased IgA and IgM level	c.3430C > T, p.Arg1144Ter	ND	ND	[16]
13	ND	Chinese	ND	ND	ND	ND	ND	ND	Leukopenia, increased CD3 +, CD4 +, and CD8 +, decreased CD19 + and NK, decreased IgM and IgG	c.5335A > T, c.2423 T > C	ND	ND	[14]
14	ND	Chinese	ND	ND	ND	ND	ND	ND	Increased CD19 +, decreased NK, increased IgA and IgG	c.743A > G, c.5048C > T	ND	ND	[14]
15	F	Iranian/Turkish	+	+	18	18	Pneumonia, otitis media, skeletal tuberculosis	Renal failure, pneumothorax, and seizure	Lymphopenia, decreased CD4 +, increased CD19 +, CD20 +,CD16 +, and CD56 +, decreased IgM, increased IgE, Non-protective against diphtheria toxoid	c.1512delG: p.I505Sfs*28	Antibiotics, antiviral, antifungal, and anti-tuberculosis drugs, peritoneal dialysis	Dead	Our case

*No*. number, *CON*. consanguinity, *FH* family history, *AOO* age of onset, *m* months, *AF-Inf* age of first infection, *Ref*. reference, *ND* not determined, *RSV* respiratory syncytial virus, *HSCT* hematopoietic stem-cell transplantation, *CMV* cytomegalovirus, *HHV* human herpes virus, *Ig* immunoglobulin, *NK* natural killer, *IVIG* intravenous immune globulin, *TRECs* T cell receptor excision circles, *PHA* phytohemagglutinin, *ARDS* acute respiratory distress syndrome, *BCG* *Bacillus* Calmette–Guérin, *FTT* failure to thrive, *VZV* Varicella-zoster virus, *HBV* hepatitis B virus, *KLH* keyhole limpet hemocyanin, *PRP* polyribosylribitol phosphate

## Discussion

In this study, we reported a 27-month-old girl presenting with recurrent, severe, early-onset infections. The laboratory data revealed defective cellular and humoral immune systems and the patient was found to harbor a novel homozygous frameshift deletion mutation c.1512delG (p.I505Sfs*28) at exon16 of the *DOCK2* gene*.* Similar to the immunological results of patients reported by *Dobbs *et al*.* and *Moens *et al*.* [3, 12], our patient had lymphopenia along with decreased CD4 + cell count and IgM level. However, in contrast to our patient’s results, these studies also described decreased CD3 + and CD8 + cell counts as well as defective antibody responses in patients with DOCK2 deficiency [3, 12]. Of note, the persistent lymphopenia observed in DOCK2-deficient patients could be due to the recurrent infections that occurred in these cases, which is more prominent in our patient who manifested with skeletal tuberculosis, an unusual infection that has not been previously reported. In contrast, the index patient in *Moens *et al*.*’s study was reported to have lymphopenia and reduced TREC level at birth and prior to infection [12]. Therefore, more studies are required to elucidate whether lymphopenia is inherent to DOCK2 deficiency. Additionally, our patients received age-matched vaccination including BCG, and did not represent any adverse reaction.

*Dobbs *et al*.* were the first study that described DOCK2-deficient patients with three out of the five patients developing HHV infections [3]. Later, *Alizadeh *et al*.* proposed a hypothesis based on the results of their patient and *Dobbs *et al*.*’s, indicating a probable association between DOCK2 deficiency and susceptibility to various HHV infections [5] which was subsequently strengthened by the results of other studies [12, 13]. Even so, no sign of HHV-related infections was detected in our case. We also observed elevated IgE level in our patient that was only expressed previously by *Dobbs K *et al*.* [3]. Unlike our patient’s laboratory data, most of the DOCK2-deficient patients had decreased CD19 + cell counts. However, a recent study of a Chinese patient also reported an increased CD19 + cell count [14].

We found a total of 14 DOCK2-deficient patients skewing toward male patients (57.1%) in the published literature. Positive family history of immunodeficiency and parental consanguinity were important factors in the patients that are affirmative according to the autosomal recessive inheritance of DOCK2 deficiency [3]. The prevalent immunological abnormalities among reported patients were as follows: low CD3 +, low CD4 +, low CD8 +, low CD19 +, and low NK cell counts along with reduced IgM and normal IgG levels. Decreased T-cell responses to PHA were also present in the range defined for SCID [11]. Given all cases with available data had low TREC levels and the arguable evidence of lymphopenia adherence to this disease, DOCK2 deficiency could be recognizable through newborn SCID screening programs [15]. Unfortunately, we were not able to determine the T-cell response to PHA and TREC levels of our patient due to her rather rapid clinical deterioration and death.

## Conclusion

In summary, DOCK2 deficiency should be contemplated in the context of severe or unusual early-onset infections, especially HHV infections, accompanied by laboratory data indicating both cellular and humoral defects. Decreased CD4 + T cell count was the most prevalent immunological abnormality detected in these patients. The role of the newborn TREC screening program in detecting suspected patients with DOCK2 deficiency needs to be clarified in the upcoming studies. Further investigations are required to discover the possible associations between this genetic defect and the laboratory/or clinical features of the disease.

## Data Availability

All data generated or analyzed during this study are included in this published article.

## Abbreviations

DOCK2: Dedicator of cytokinesis 2
Ig: Immunoglobulin
HHV: Human herpes virus
NK: Natural killer
ATP: Adenosine triphosphate
ROS: Reactive oxygen species
CID: Combined immunodeficiency
PIDTC: Primary immunodeficiency treatment consortium
MTB: Mycobacterium tuberculosis
TRECs: T cell receptor excision circles
PHA: Phytohemagglutinin
HSCT: Hematopoietic stem cell transplantation
SCID: Severe combined immunodeficiency
WES: Whole-exome sequencing
PICU: Pediatric intensive care unit
CMV: Cytomegalovirus
